# Aberrant Development of Thymocytes in Mice Lacking Laminin-2

**DOI:** 10.1155/2000/90943

**Published:** 2000

**Authors:** William J. Magner, Andrew C. Chang, Jennie Owens, M-J. P. Hong, Andrew Brooks, John E. Coligan

**Affiliations:** 1 Laboratory of Allergic Diseases NIAID NIH Rockville MD 20852-1727 USA; 2 Division of Hematology CBER FDA USA; 3 CLaboratory Sciences Section NCRR NIH Bethesda MD 20892 USA; 4 Roswell Park Cancer Institute Buffalo NY 14263 USA

**Keywords:** apoptosis, dystrophic, integrins, laminin, thymocytes

## Abstract

In previous *in vitro* studies, we proposed a role for the extracellular matrix component, laminin- 2, and its integrin receptor, VLA-6, in thymocyte development. The characterization of two dystrophic mouse strains with different defects in laminin-2 allowed us to examine this proposal *in vivo*. Mice deficient in laminin-2, *dy*/*dy*, show a significant reduction in thymus size and number of thymocytes compared to normal littermates. These mice also exhibited apparent alterations of thymic architecture. Examination of the CD4/CD8 populations in *dy*/*dy* thymi showed large relative increases in the DN (CD4^-^CD8^-^) and SP (CD4^+^CD8^-^, CD4^-^CD8^+^) populations and a significant decrease in the DP (CD4^+^CD8^+^) population. Further examination of the DN population for CD44 and CD25 expression showed a remarkable decrease in the more mature pre-T cell populations. Analysis of apoptosis *in situ*, and by flow cytometry, in *dy*/*dy* thymi revealed a significant increase in apoptotic DN thymocytes in the capsule and subcapsular regions. Interestingly, thymocyte development appeared to proceed normally in dystrophic mice expressing a mutant form of laminin-2, *dy*^2J^, as well as, in fetal and neonatal *dy*/*dy* mice. We propose that laminin-2 plays an active role in thymocyte development by delivering cell survival and differentiation signals at specific stages of development in young adult mice.


*Developmental Immunology*, 2000, Vol. 7(2-4), pp. 179-193
Reprints available directly from the publisher
Photocopying permitted by license only

© 2000 OPA (Overseas Publishers Association)
N.V. Published by license under the
Harwood Academic Publishers imprint,
part of the Gordon and Breach Publishing Group.
Printed in Malaysia

### Aberrant Development of Thymocytes in Mice Lacking Laminin-2

WILLIAM J. MAGNER[a*†], ANDREW C. CHANG[b], JENNIE OWENS[c], M-J. P. HONG[a], ANDREW BROOKS[a] and JOHN E. COLIGAN[a‡]

[a]*Laboratory of Allergic Diseases, NIAID, NIH, Rockville, MD 20852–1727,* [b]*Division of Hematology, CBER, FDA and* [c]*Laboratory Sciences Section, NCRR, NIH, Bethesda, MD 20892*

In previous *in vitro* studies, we proposed a role for the extracellular matrix component, laminin-2, and its integrin receptor, VLA-6, in thymocyte development. The characterization of two dystrophic mouse strains with different defects in laminin-2 allowed us to examine this proposal *in vivo*. Mice deficient in laminin-2, *dy/dy*, show a significant reduction in thymus size and number of thymocytes compared to normal littermates. These mice also exhibited apparent alterations of thymic architecture. Examination of the CD4/CD8 populations in *dy/dy* thymi showed large relative increases in the DN ($CD4^-CD8^-$) and SP ($CD4^+CD8^-$, $CD4^-CD8^+$) populations and a significant decrease in the DP ($CD4^+CD8^+$) population. Further examination of the DN population for CD44 and CD25 expression showed a remarkable decrease in the more mature pre-T cell populations. Analysis of apoptosis *in situ*, and by flow cytometry, in *dy/dy* thymi revealed a significant increase in apoptotic DN thymocytes in the capsule and subcapsular regions. Interestingly, thymocyte development appeared to proceed normally in dystrophic mice expressing a mutant form of laminin-2, $dy^{2J}$, as well as, in fetal and neonatal *dy/dy* mice. We propose that laminin-2 plays an active role in thymocyte development by delivering cell survival and differentiation signals at specific stages of development in young adult mice.

*Keywords:* apoptosis, dystrophic, integrins, laminin, thymocytes

### INTRODUCTION

The laminins are a family of extracellular matrix (ECM) proteins found in basement membranes; they are heterotrimeric proteins consisting of a heavy chain (α) and two light chains (β, γ). Laminin-1 (classically known as laminin) is composed of the α1, β1 and γ1 chains while laminin-2 (merosin) contains the α2, β1 and γ1 chains (Wewer and Engvall 1994, Burgeson et al, 1994). Laminin-2 has been shown to be a ligand for VLA-6 ($\alpha_6\beta_1$), an integrin which is expressed on thymocytes (Chang et al, 1993, Lannes-Vieira et al, 1993). The three component chains of laminin self-assemble to form the mature laminin molecule which then polymerizes and represents a major component of the basal lamina (Lannes-Vieira et al, 1990). The various laminin isoforms are expressed in a tissue or developmental

* First two authors contributed equally to this publication.
† Current address: Roswell Park Cancer Institute, Buffalo, NY 14263.
‡ Corresponding author: John E. Coligan, Ph. D., NIH/NIAID, Twinbrook II, Rm. 205, 12441 Parklawn Dr. Rockville, MD 20852–1727. voice 301–496–8247, FAX 301–480-9094, jcoligan@niaid.nih.gov stage – specific manner (Leivo and Engvall, 1988, Ehrig et al, 1990, Wewer et al, 1994).

During thymocyte development, maturation and selection, the thymocytes migrate through the different compartments of the thymus (Sprent et al, 1988, Scollay and Godfrey, 1995). Each compartment presents a different microenvironment to the developing thymocytes and directly affects the differentiation, proliferation and survival of the cells passing through it (Ritter and Boyd, 1993). The earliest populations of thymocytes are $CD4^-CD8^-$ cells referred to as "double negative" (DN). This population may be subdivided into several defined developmental populations by a variety of markers including CD44 and CD25 (Godfrey et al, 1993, Godfrey et al, 1994). As double negative cells mature, they acquire CD3, CD4 and CD8 surface expression as they progress to a population referred to as double positive ($CD4^+CD8^+$) (DP). The double positive cells further mature along either the CD4 or CD8 pathway to mature single positive cells (for a review see Guidos, 1996). By the time mature single positive thymocytes exit the thymus for the periphery, they will have undergone positive and negative selection -- processes which delete self-reactive cells and provide self-MHC-restricted immunocompetent effector T cells to the periphery.

Laminin-2 has been shown to be expressed in the adult mouse thymus exclusive of laminin-1 and certain immature populations of murine thymocytes ($J11d^+$) have been shown to adhere to laminin-2 (Chang et al, 1993). This evidence, together with previous data (Wadsworth et al, 1993), is suggestive of a role for laminin-2 in thymocyte development.

Various cells recognize and bind to extracellular matrix molecules through cell surface receptors of the integrin family. The numerous heterodimeric integrin molecules are classified according to the β chain used; each β chain can associate with between one and nine different α chains. Thymocytes express several β1 integrins (Wadsworth et al, 1993) including the laminin-2 receptor, α6β1 (VLA-6). Numerous studies have examined the functions of the integrins (Hynes, 1992, Giancotti, 1997, Savino and Silva-Barbosa, 1996, Malik, 1997) and two groups have produced β1 knockout mice (Stephens et al, 1995, Fassler and Meyer, 1995). Both of these groups found that disruption of β1 function led to embryonic lethality. The *dy* (dystrophia muscularis) mice, in which laminin-2 expression is undetectable, have provided a "knockout" system with which to investigate the possible role that laminin-2 may play in T cell ontogeny.

Muscular dystrophy is an autosomal recessive, neuromuscular degenerative disease, characterized by muscle degeneration and dysmyelination in the nervous system (Duowitz, 1992, Worton, 1995, Matsumura and Campbell, 1993). The *mdx* and *dy* mice are two of the animal models used in the study ofthis disease (Campbell, 1995). The dystrophin gene was cloned and found to be deficient in the *mdx* mouse but normal in the *dy* mouse (Hynes, 1992, Worton, 1995, Xu et al, 1994a). The defect in the homozygous *dy* mouse (*dy/dy*) has been identified as a lack of laminin-2, an anchor protein in basal lamina important for muscle cell binding. It has been hypothesized that disruption of the linkage between the subsarcolemmal cytoskeleton and the basal lamina could result in the muscle cell necrosis and peripheral neuropathy characteristic of the *dy/dy* mouse and human muscular dystrophy patients (Sunada et al, 1994, Xu et al, 1994a, Xu et al, 1994b). The $dy^{2J}$ mouse, which expresses a form of murine dystrophia muscularis related to but distinct from the *dy* strain, was also analyzed to determine its genetic defect (Xu et al, 1994a, Sunada et al, 1995). These mice have near normal levels of laminin-2 expression in their tissues but they display symptoms comparable to the *dy/dy* mice. In the $dy^{2J}$ strain, the laminin α2 chain gene was shown to carry a mutation identified as a 171 bp in-frame deletion as well as a splice donor site mutation in the laminin-2 heavy chain transcript. The expressed form of the α2 chain in these mice has a 57 amino acid deletion (residues 34–90) and a substitution (Q91E) in the N-terminal domain VI (Xu et al, 1994a, Sunada et al, 1995). These mutations are sufficient to mimic the muscular dystrophy phenotype of mice lacking laminin-2 expression.

We have taken advantage of the laminin-2 deficiency in the *dy/dy* mice to examine the role of laminin-2 in thymocyte development. We show that the *dy/dy* thymus is reduced in size out of proportion to the overall diminished size of the animals and that the thymic architecture is dramatically altered, especially in the reduction of the cortical regions. This diminished cortex correlated with a dramatic reduction in the size of the $CD4^{+}CD8^{+}$ (DP) population, normally the largest population in the thymus. The *relative* size of the $CD4^{-}CD8^{-}$ population was markedly increased while the more mature subpopulations within this subset were reduced in size. The subpopulations that were relatively reduced in size in the *dy/dy* mice were the populations that show the highest affinity for laminin-2. We present a model for the role of laminin-2 in thymocyte development and immunohistochemical analyses of *dy/dy* and *dy* control thymi to support our conclusion that a likely cause of these developmental deficiencies is an abnormal level of apoptotic thymocyte death We believe this death results from the lack of a differentiation or survival signal that is normally provided to immature thymocytes by laminin-2.

### MATERIALS AND METHODS

### Mice

Adult (4–6 week old) C57BL/6J-*dy* male and female mice, with control littermates, were obtained from The Jackson Laboratory, Bar Harbor, ME. C57BL/6J-$dy^{2J}$ male mice were a generous gift from Dr. Eva Engvall of The Burnham Institute (La Jolla Cancer Research Foundation), La Jolla, California. Homozygous *dy/dy* mice can not breed; heterozygous mice were bred and homozygous *dy/dy* mice selected from the litters by phenotype. Heterozygous dystrophic mice (*dy*/+) were not distinguished from wild type (+/+) therefore, control littermates will be referred to as *dy* control mice (+/?) throughout this paper; the homozygous mutant mice are referred to as *dy/dy*. The C57BL/6J-$dy^{2J}$ mice used were all homozygous for the mutation and will simply be referred to as $dy^{2J}$.

### Antibodies

The antibodies used for FACS analyses -- anti-CD25 (clone 7D4 labeled with FITC), anti-CD44 (clone IM7 labeled with PE), anti-CD4 (clone RM-4–5 labeled with PE), anti-CD8 (clone 53–6.7 labeled with FITC) -- were purchased from Pharmingen, San Diego, CA.

The antibodies used for immunofluorescent staining were anti-laminin clone I (purchased from Life Technologies Inc., Gaithersburg, MD) and the anti-medullary epithelia (ERTR5) which was generously provided by Dr. Willem van Ewijk, Dept. of Immunology, Erasmus University, Rotterdam, The Netherlands. Cortical thymic epithelia were stained with the monoclonal rat anti-mouse antibody (IgM) clone 4F1 (BioSource International, Camarillo, CA).

The antibodies used in ELISA analyses of ECM extracts were mouse anti-human laminin-2, which is known to cross-react with mouse laminin-2 (Chemicon International Inc., Temecula, CA), rabbit anti-mouse fibronectin (Life Technologies, Inc., Gaithersburg, MD) and rabbit anti-mouse laminin (Upstate Biotechnology Inc., Lake Placid, NY).

The antibodies used for immunodepletion were 3.155 anti-CD8 antibody (TIB-211 hybridoma) and GK1.5 anti-CD4 antibody (TIB-207 hybridoma) from ATCC, Rockville, MD.

### Cells and Tissues

Adult thymocytes were prepared as described (Chang et al, 1993). Briefly, thymi were gently minced between two layers of fine mesh nylon screen in Medium 199 (Life Technologies, Inc., Gaithersburg, MD) with 0.5% BSA and the cell suspensions were filtered through nylon to remove debris.

$CD4^{-}CD8^{-}$ thymocytes were prepared by complement (Cedarlane Laboratories, Ltd., Hornby, Ontario, Canada) mediated depletion with 3.155 anti-CD8 monoclonal antibody followed by DynaBead (Dynal Inc., Lake Success, NY) removal of $CD4^{+}$cells with GK1.5 monoclonal antibody coated sheep anti-rat dynabeads according to manufacturers instructions.

Mouse thymus samples were fixed in 10% neutral phosphate-buffered formalin, embedded in paraffin, and sectioned at 6μm before staining with hematoxylin-eosin (H&E) by standard methods.

TABLE I Analysis of dystrophic mouse mass and cell numbers[a,b]

| *genotype* | *body weight (g) ± SD* | *thymus weight (mg) ± SD* | *thymus wt (mg)/body wt (g)* | *cells/thymus ± SD* $(\times 10^{-6})$ | *cells/mg thymus* $(\times 10^{-6})$ |
|---|---|---|---|---|---|
| *dy* control | 25.9 ± 1.3 | 42.8 ± 11. | 1.7 | 67.8 ± 23.3 | 1.6 |
| *dy/dy* | 10.9 ± 1.4 | 10.6 ± 8.3 | 1.0 | 7.5 ± 4.72 | 0.7 |

[a.] Dystrophic mice with a genetic defect in the expression of laminin-2 were compared to *dy* control mice on the basis of physical characteristics of thei bodies and thymi; all mice were matched for age and sex. Thymi from *dy/dy* mice demonstrate a dramatic reduction in thymocyte number.
[b.] Each value is the average of three determinations.

For immunohistochemical analyses, thymus tissues were snap-frozen in Tissue-Tek O.C.T. compound chilled with methylbutane/dry ice. Frozen sections were prepared with a Tissue-Tek cryostat microtome. The sections were air-dried, fixed in acetone at –20 °C for 10 min, preincubated in 10% normal goat serum to prevent non-specific binding of antibodies, and then incubated at 37 °C for 60 min with primary antibodies diluted in 2% goat serum. After washing with phosphate-buffered saline, species-appropriate fluorescein-conjugated secondary antibodies were applied for 45 min. The slides were examined under an Olympus BH-2 fluorescent microscope. Replacement of primary antibodies with either IgM or IgG served as a negative control for each antibody. Positive control sections of normal mouse thymus tissues were also tested.

### Flow Cytometry

Flow cytometric analyses were conducted by standard methods on a FACSort (Becton Dickinson, San Jose, CA). Data presented are representative of at least three independent experiments. Each two-dimensional dot plot represents 10,000 cells analyzed.

Flow cytometric analysis of apoptosis was accomplished through use of Beckman-Coulter flow cytoenzymology technology. The CellProbe reagents YVAD-Caspase 1, DEVD-Caspase 3 and VEID-Caspase 6 were generously supplied by Dr. Frank Lucas of the Coulter Corporation (Miami, Florida). These reagents incorporate a tetrapeptide substrate specific to each of the caspase enzymes with a fluorophore C terminal to the proteolytic cleavage site. The assays were conducted as described by Coulter; basically, cells are prepared by standard means for flow cytometry then, 60 minutes prior to analysis, the substrate is added to the cell suspension. The substrate is freely permeable to the cell membrane. Flow cytoenzymology, simultaneous with typical flow cytometry, was carried out on a FACS Calibur (Becton Dickinson, San Jose, CA). Caspase activity was quantitated on FL1, surface markers on FL2 and FL4 and propidium iodide exclusion was monitored on FL3.

### Cell Adhesion Assay

Thymocyte binding to ECM proteins was assayed as described by Shimizu *et al.* (Shimizu et al, 1990) with minor modifications. Briefly, aliquots of 100 µl of 10 µg/ml of human laminin-2 or mouse fibronectin in PBS (containing $Ca^{++}$ and $Mg^{++}$) were added to 96-well flat-bottom microtiter plates (Removawell Strip System, Dynatech Laboratories, Inc., Chantilly, VA) and incubated at 4 °C overnight. After washing the wells and blocking with 2.5% BSA in PBS for 2–2.5 h at 37 °C, $^{51}$Cr-labeled cells ($5 \times 10^5$/well) were added in 100 µl of assay medium (Medium 199 with 0.5% BSA). After 45 min at 4 °C, plates were rapidly warmed to 37°C for 15 min; nonadherent cells were removed by gently washing three times with the assay medium. Wells were then separated and bound radioactivity was determined by gamma counting. Data are expressed as the mean percent of cells bound in triplicate wells. Percent cells bound was calculated as (bound cpm/total cpm added per well) × 100. Background binding to BSA-coated wells was 1–2% and was not subtracted from the data.

### Analysis of Apoptosis in situ

Tissue sections (3 μm) were analyzed for apoptosis by TUNEL (*T*dT-mediated d*U*TP *N*ick *E*nd *L*abeling) staining. These analyses were conducted by Chesapeake Histology Labs, Inc., Baltimore, MD according to the method of Brown *et al* (Brown et al, 1996).

### Results

### *Quantitation of Thymocytes in Dystrophic Mice*

The *dy/dy* mice display classic muscular dystrophy symptoms including muscle weakness and impaired motor abilities. The *dy/dy* mice are apparent from their inability to fight when held by their tails, splayed limbs, difficulty of movement, small body size and lack of muscle mass, relative to heterozygous and wildtype littermates. The differences in body and organ size between *dy/dy* mice and their normal (*dy*/+ and +/+) littermates were quantitated for the purposes of comparison and control within our analyses (Table I). In this set of data, the decreased size of the *dy/dy* mice is apparent, as is the diminished mass of their thymi. Thymus weight and the number of thymocytes recovered from each thymus are standardized to body mass and thymus mass respectively to demonstrate that the thymus is reduced in size and cell numbers even relative to the reduced body size of these mice, i.e. the loss of thymocytes is out of proportion with the size of the mice. Interestingly, in the C57BL/6J-$dy^{2J}$ mice, which display neuromuscular symptoms comparable to the *dy/dy* mice, the size of the thymus and the number of thymocytes recovered were similar to the *dy* control littermates (data not shown). These results indicate that expression of laminin-2 is required for normal thymic development and that the N-terminal truncated laminin-2 expressed by the $dy^{2J}$ mice is sufficient to provide for normal development.

### *Analysis of Dystrophic Thymic Architecture*

Having observed the dramatically decreased mass of the thymus and the loss of thymocytes in the *dy/dy* mice, histological analyses were carried out to examine the structure of the thymus, its component cell types and the expression of extracellular matrix components in the thymus.

To examine the *dy/dy* thymic architecture, hemotoxylin-eosin (H&E) stainings were performed. Comparison of H&E staining of the *dy/dy* thymus with a control littermate thymus showed not only the decreased size of the dystrophic thymus, but also the abnormal thymocyte distribution within the dystrophic thymus (Fig. 1A). The difference in size between the *dy/dy* and *dy* control thymi is apparent in this figure where, at the same magnification, the *dy* control photomicrograph shows a portion of a thymus while the *dy/dy* photomicrograph shows an entire thymus. The cortical and medullary regions of the *dy/dy* thymus cannot be distinguished by H&E staining. The cell density throughout the *dy/dy* thymus is comparable to the cell density in the medullary region of the *dy* control thymus (Fig. 1A). This apparent diminution of the cortex may be explained by destruction of thymic epithelium or by loss of cortical thymocytes.

To examine the distribution of thymic epithelial cell types, thymic sections were stained with antibodies specific for cortical or medullary epithelia. Figure 1B shows the staining pattern with the ERTR5 anti-medullary epithelium and Figure 1C shows the staining with 4F1 anti-cortical epithelium antibodies. It is apparent from these stainings that the cortical and medullary epithelia are present in the *dy/dy* thymi. Therefore, the apparent loss of the cortex in these mice must mainly result from a diminution in cortical thymocytes.

These thymic sections were also analyzed for expression of the laminin chains which are common to both laminin-1 and laminin-2. Figure 1D shows the immunofluorescent staining pattern obtained with the Clone I antibody which is specific for the laminin β1 and γ1 chains. Expression of the laminin β1 and γ1 chains can be detected in both thymic cortical and medullary regions from *dy* control mice. It is clear that expression of these chains is lost in the *dy/dy* thymi.

### *Flow Cytometric Analysis of Thymocyte Populations in Dystrophic Mice*

Since it appeared that the *dy/dy* mice demonstrated a lack of cortical thymocytes, we performed a variety of

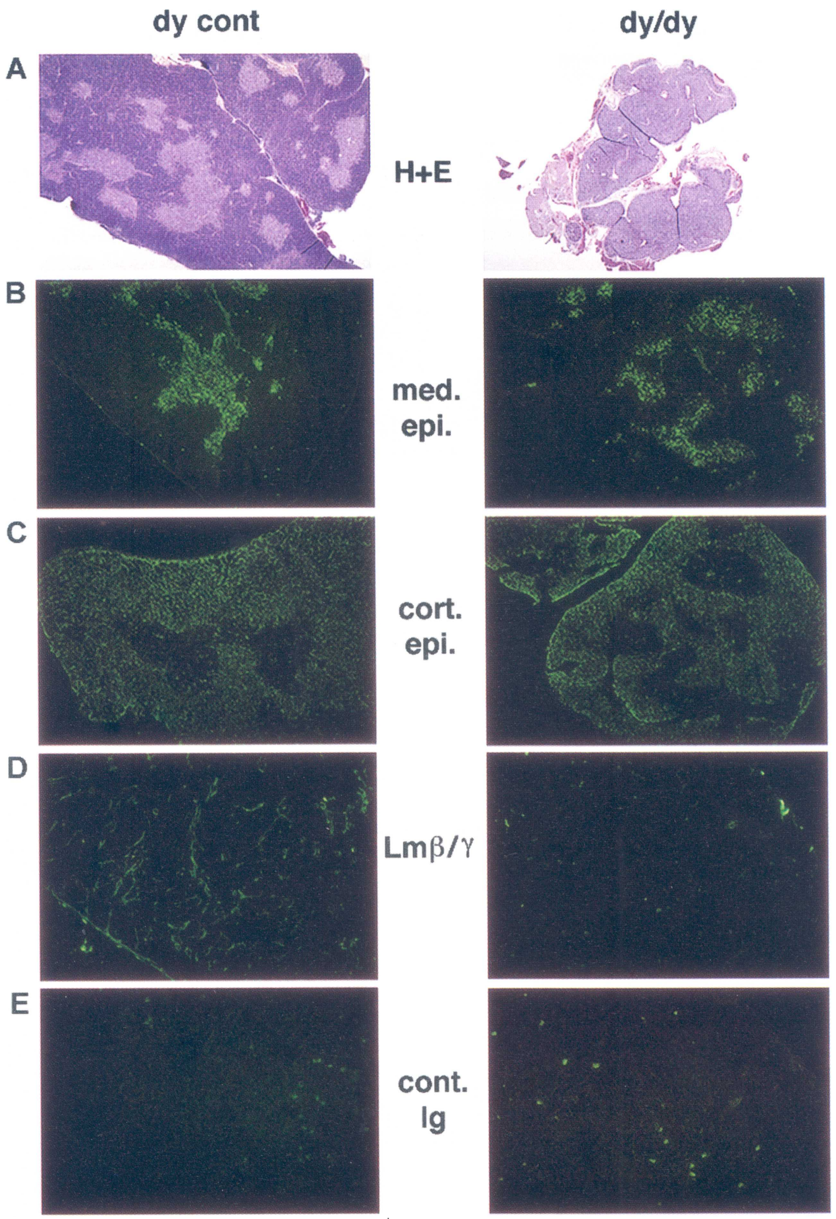

FIGURE 1 Immunohistochemical analyses of *dy/dy* and *dy* control thymi. Panel A is shown at 25X magnification, panels B-E are 50X. A Hemotoxilin-Eosin staining of thymic sections were performed to reveal the intact thymic architecture. As stated in Table I, the *dy/dy* thymi are much smaller and contained fewer cells than those of control littermates. The cortex (C) and medulla (M) are clearly visible in the *dy* control but not in the *dy/dy* thymus. B & C. Immunohistochemical staining for cortical and medullary epitopes. To characterize the thymic architecture, thymic sections were stained with antibodies specific for cortical or medullary epithelia (4F1 and ERTR5 respectively). The cortical and medullary epithelia are clearly present but smaller or more compact in the *dy/dy* mice. D. Immunohistochemical staining for laminin β1 and γ1 chains. To examine laminin expression, thymic sections were stained with the Clone I antibody which is specific for the laminin β1 and γ1 chains; these chains are common to laminin-1 and laminin-2. The *dy/dy* thymus is clearly deficient in laminin β1 and γ1 protein expression. The antibody used is IgM, the high background appeared to be characteristic of this antibody. Despite the high background, the classic ECM network staining is not visible in the *dy/dy* section. E. Isotype matched negative control for Figure 1D (see Color Plate XII at the back of this issue)

flow cytometric analyses to examine discrete populations of developing thymocytes. Unfractionated adult thymocytes were stained for two-color flow cytometry with anti-CD4 and anti-CD8 antibodies for analysis of double negative, double positive and single positive populations. A representative plot of CD4/CD8 analysis is shown in Figure 2A. The proportion of all populations was significantly larger in the *dy/dy* mice except for the DP thymocyte population which was significantly smaller (83 % vs. 33 %). The four-fold increase in mature thymocytes (CD4$^+$CD8$^-$, CD4$^-$CD8$^+$) in *dy/dy* mice relative to control mice (44% vs 12% of total thymocytes) (Fig. 2A) may, at least partially, explain the diminished cortical regions of *dy/dy* mice. All of the thymocyte populations examined in the *dy*$^{2J}$mice were comparable to wild type C57BL/6J as a percentage of total thymocytes and absolute cell numbers (CD4/CD8 analysis is shown in Figure 2A). These results suggest that the loss of thymocyte populations observed in the *dy/dy* mice was specific to the lack of laminin-2 in their thymi, was not a secondary effect of their physical condition since *dy*$^{2J}$ mice, which display neuromuscular symptoms comparable to the *dy/dy* mice, had normal thymocyte populations, and was not dependent on the N-terminus of the laminin α2 chain.

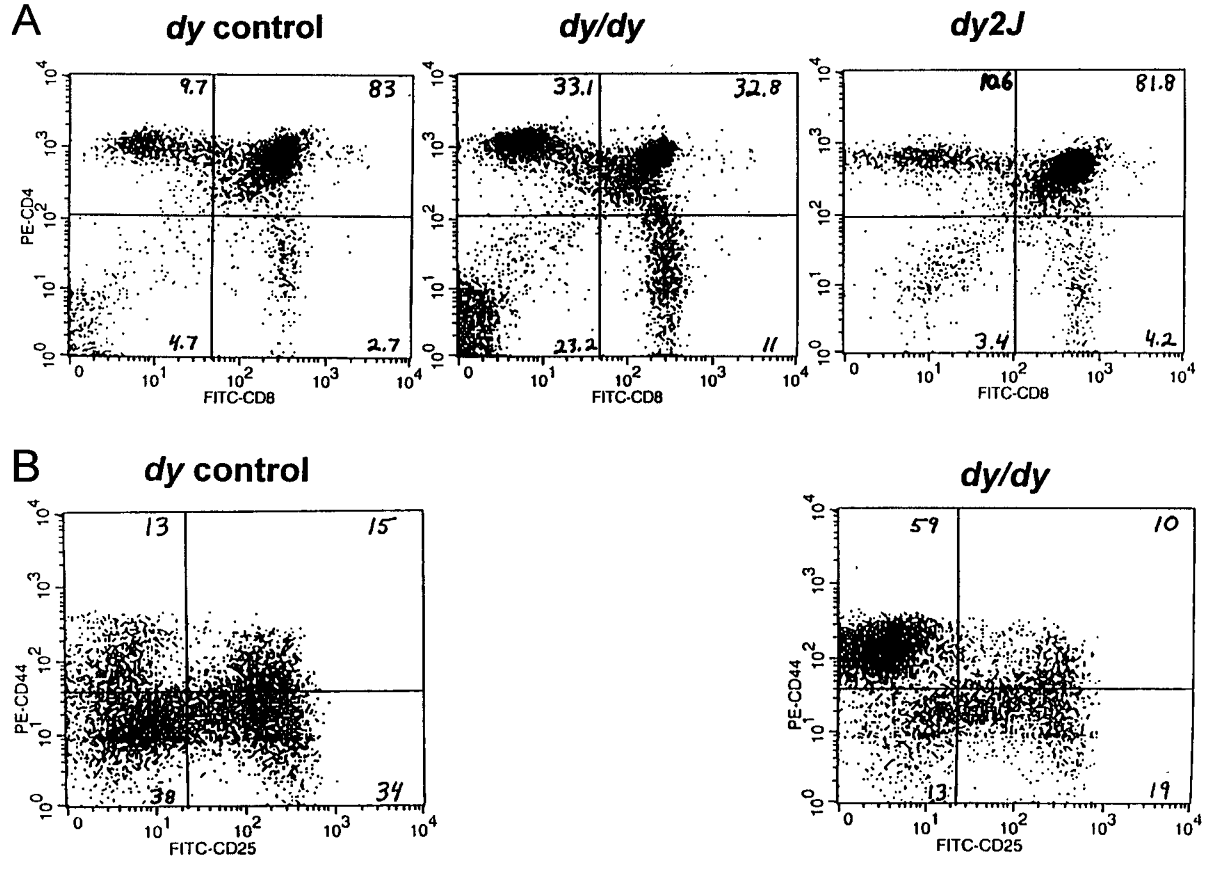

FIGURE 2 Flow cytometric analyses of dystrophic and control thymocytes. To characterize thymocyte populations, thymocytes isolated from dystrophic and control mice were stained with antibodies to thymic subset markers and analyzed by flow cytometry. The quadrant percentages are shown within each quadrant. These plots clearly show significant loss of cells from the CD4$^+$CD8$^+$and CD4$^-$CD8$^-$CD44$^-$CD25$^+$ populations in the *dy/dy* mice. A. Bulk thymocytes from *dy/dy* and *dy* control and *dy*$^{2J}$ mice were stained with PE-anti-CD4 and FITC-anti-CD8. Maturation progresses from CD4$^-$ CD8$^-$ to CD4$^+$CD8$^+$ to CD4$^+$CD8$^-$ or CD4$^-$CD8$^+$ B. DN thymocytes were prepared from *dy/dy* and *dy* control animals and stained with PE-anti-CD44 and FITC-anti-CD25. Maturation progresses from CD44$^+$CD25$^-$ to CD44$^+$CD25$^+$ to CD44$^-$CD25$^+$ to CD44$^-$CD25$^-$

The fact that the least mature CD4/CD8 (DN) thymocyte subpopulation showed such a dramatic proportional increase in *dy/dy* versus *dy* control mice (23% vs 5%) led us to examine whether developmental defects could be detected in this population. To examine early thymocyte development in *dy/dy* mice, adult thymocytes were fractionated and the double negative population was recovered. These cells were stained with anti-CD44 and anti-CD25 antibodies for analysis of the developing early thymocyte populations. The most immature thymocytes examined (Figure 2B) are the $CD44^{+}CD25^{-}$ population. A diminution of this population could indicate a defect in the ability of thymocyte progenitors to home to the thymus (Ruiz et al, 1995). In this case however, the relatively large number of DN $CD44^{+}CD25^{-}$ thymocytes in *dy/dy* mice makes a homing defect unlikely but, instead, the relative four-fold increase of this population in *dy/dy* mice as compared to *dy* control mice (59% vs 13%), makes it more likely that many of the immature thymocytes fail to receive a proper differentiation signal. Once escaping this stage of differentiation, development appears to proceed normally as the ratio of cells in the progressively more mature $CD44^{+}CD25^{+}$, $CD44^{-}CD25^{+}$, $CD44^{-}CD25^{-}$ cell compartments is about the same for *dy/dy* and *dy* control mice. These studies indicate that, in *dy/dy* mice, cells tend to accumulate within the most immature thymocyte subpopulation indicating that many fail to receive a signal necessary for differentiation. Whether all immature thymocytes require this differentiation signal and/or whether the cells that do differentiate do so normally are questions for examination.

### *Ability of dy/dy Thymocytes to Adhere to Laminin-2*

Since thymocytes of *dy/dy* mice develop in the absence of laminin-2, we wanted to determine whether these thymocytes maintain their ability to bind to laminin-2 or whether the thymocytes present in the *dy/dy* mice develop from a population which can not adhere to laminin-2.

To measure the ability of *dy/dy* thymocytes to adhere to laminin-2 relative to thymocytes from wild type control mice, adhesion assays were performed on purified ECM proteins. Unfractionated thymocytes from *dy/dy*, *dy* control and $dy^{2J}$ mice were assayed for adhesion to laminin-2 and to fibronectin (Figure 3). When the wells were coated with fibronectin, thymocytes from all three mouse strains behaved equivalently, each displaying comparable, saturable binding with a maximum of approximately 15% of cells bound. When the wells were coated with laminin-2, *dy/dy* thymocytes bound best and thymocytes from the *dy* control mice displayed the weakest binding at every ECM concentration tested. We can only conclude with confidence that all three strains are capable of binding laminin-2 and that *dy/dy* thymocytes retain the ability to bind laminin-2. We have previously shown (Chang et al, 1993), through antibody treatment of thymocytes before the adhesion assay, that thymocyte binding to laminin-2 is VLA-6 dependent and binding to fibronectin is dependent upon VLA-4 and 5. The enhanced binding *dy/dy* thymocytes display toward laminin-2 is not unexpected – flow cytometric analyses demonstrated that VLA-6 expression was increased on these cells (data not shown). It is possible that thymocyte adhesion to laminin-2 leads to downregulation of VLA-6. Adhesion to laminin-2 would provide a needed co-stimulatory or survival signal followed by VLA-6 downregulation and continued migration and development. These possibilities will be the subjects of future studies.

### *Analysis of Apoptosis in Dystrophic Thymi*

The decreased cell numbers in *dy/dy* thymi could result from a lack of proliferation or from an increased death of early thymocytes. Apoptosis is a very active process in the thymus. The majority of developing thymocytes die during selection and never reach the periphery. To examine apoptosis in the *dy* mice, fixed thymi were sectioned and stained by the TUNEL method. As shown in Figure 4, the *dy/dy* thymus contains a significantly increased number of apoptotic cells relative to the *dy* control section. These apoptotic thymocytes were found in the capsular and subcapsular regions where DN thymocytes reside. The $dy^{2J}$ mice were also analyzed for thymic apoptosis. As seen in Figure 4, the $dy^{2J}$ thymi appear quite normal and do not display the increased apoptosis seen in the *dy/dy* thymi. Thus, the lack of lam-

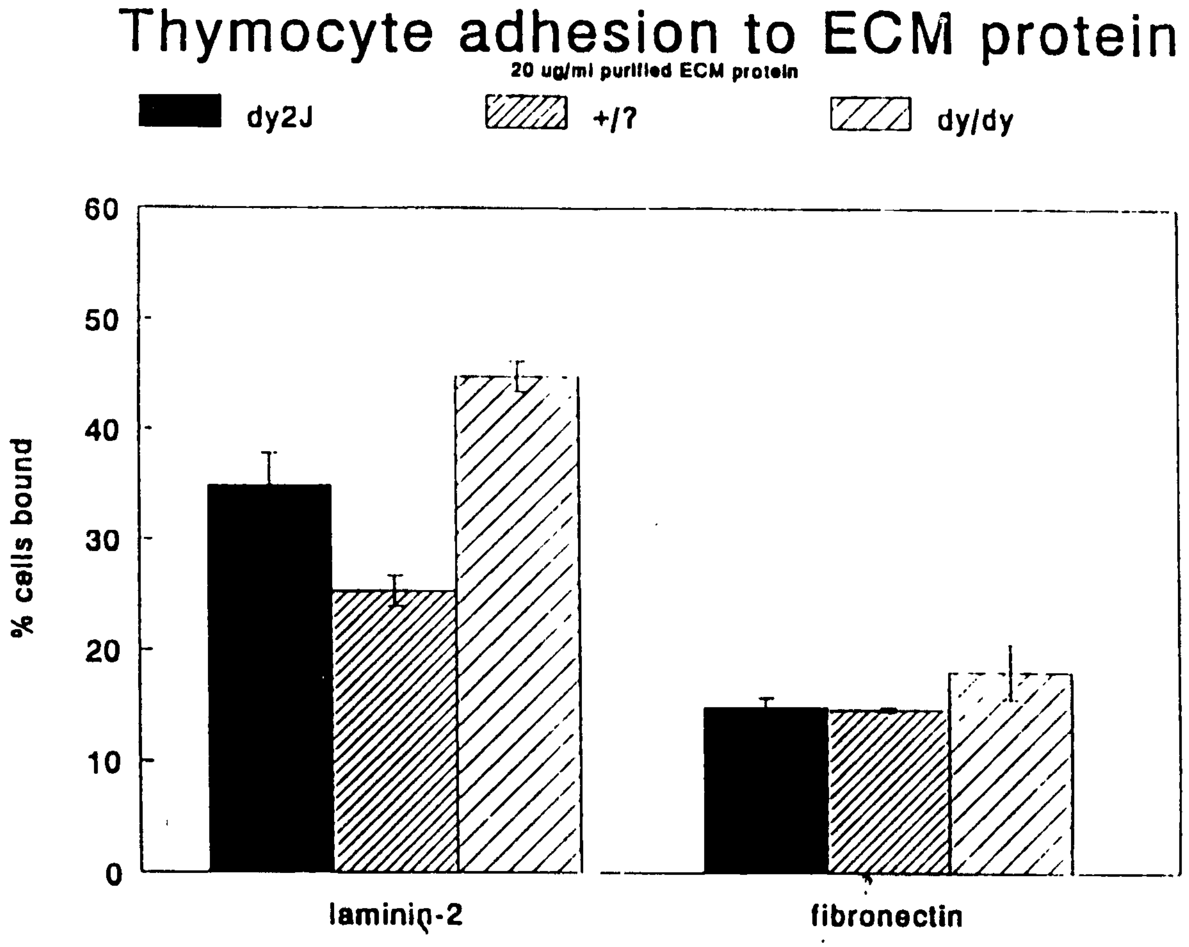

FIGURE 3 Thymocyte adhesion to ECM proteins. To analyze the ability of thymocytes from *dy/dy*, $dy^{2J}$ and *dy* control animals to bind to specific ECM proteins, adhesion assays were conducted on laminin-2 and fibronectin. The assays shown in this figure were conducted with a concentration (20 μg/ml ECM protein) used to coat the wells of the plates which provided maximal binding on each substrate inin-2 expression in *dy/dy* mice appears to result in an increased level of apoptotic cell death among early thymocytes which is not seen in $dy^{2J}$ dystrophic mice.

To identify the specific immature thymocyte population(s) which undergo enhanced apoptosis in *dy/dy* mice, flow cytoenzymology was applied in conjunction with flow cytometry. This technique allowed fresh thymocytes, surface labeled for flow cytometry, to be loaded with a substrate specific for an individual enzyme expected to be active in the apoptotic cascade (Porter and Janicke, 1999, Berliner et al, 1997). Cleavage of the substrates used in this analysis by their specific caspase enzymes, releases fluorescent molecules which are observed and quantitated via standard flow cytometry. Figure 5A illustrates the fluorescent shifts which can be achieved with this technique and serves at a control from which bounds are defined for quantitation of apoptotic cells. Through this technique, we were able to detect elevated apoptosis throughout the immature *dy/dy* thymic subpopulations but primarily among the $CD25^+$ DN thymocyte populations (Figure 5B).

The *in situ* TUNEL assay and flow cytoenzymology technique provided mutually supportive evidence

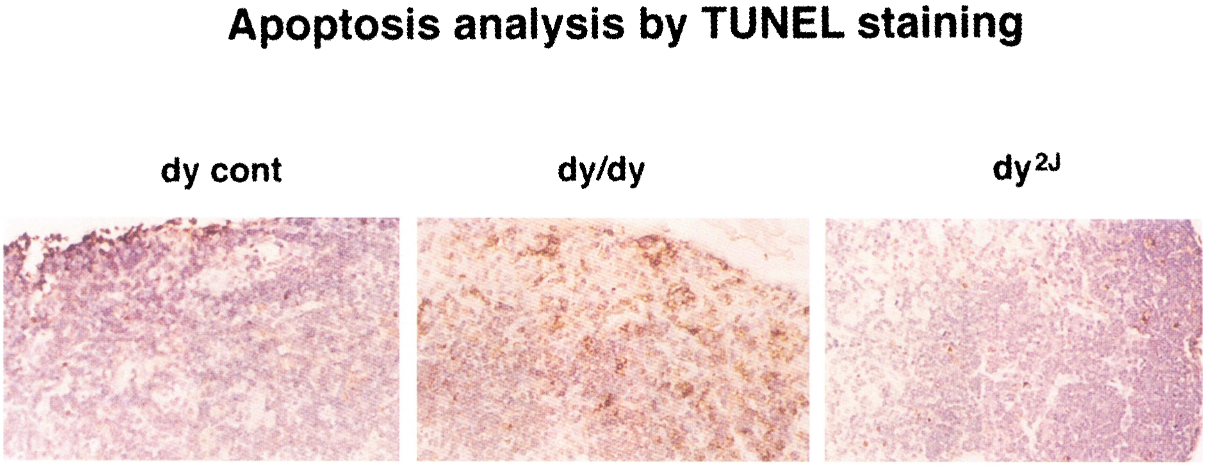

FIGURE 4 Histochemical analysis for apoptotic cells. To analyze apoptotic cell death in dystrophic mice, the TUNEL staining technique was applied to sections of thymi from dystrophic and control mice. The subcapsular region of the *dy/dy* thymus clearly contains significantly more apoptotic cells than the section from its control littermate. This supports our hypothesis of increased apoptotic death in an early thymocyte population. TUNEL staining of the $dy^{2J}$ thymi and their wildtype controls did not show any visible difference in apoptosis in the $dy^{2J}$ (see Color Plate XIII at the back of this issue)

that the $CD4^-CD8^-$ early thymocytes of *dy/dy* mice undergo a high level of apoptotic cell death. These data provide an explanation for the loss of immature thymocytes (apoptotic death) and of $CD4^+CD8^+$ thymocytes (death of their precursor population) seen in our FACS analyses of *dy/dy* mice.

### DISCUSSION

We previously showed that laminin-2, and not laminn-1, is expressed in the thymus and that a portion of thymocytes can adhere to laminin-2 (Chang et al, 1993). We also demonstrated that immobilized laminin-2 provides a co-stimulatory signal for anti-CD3-induced human thymocyte proliferation (Chang et al, 1995) These observations led to our proposal that laminin-2 plays a specific role in the development of thymocytes. Thus, we have used the laminin-2 deficient *dy* mouse as a model system in which to examine this hypothesis.

The thymi of these dystrophic mice are disproportionately reduced in mass when compared to their control littermates. The number of thymocytes which can be isolated from these thymi is also reduced out of proportion with the size of the thymus. Phenotypic analysis of these thymocytes for CD4 and CD8 expression showed that the proportion of DN cells was dramatically increased in *dy/dy* mice along with a concomitant decease in DP cells. This suggested that many immature (DN) thymocytes were failing to develop properly. Examination of the $CD4^-CD8^-$ population for expression of the CD44 and CD25 developmental markers revealed a large proportional increase in the most immature thymocyte population ($CD44^+CD25^-$) in *dy/dy* mice (Fig. 2B). Thus, at the earliest stage of maturation, many *dy/dy* thymocytes fail to receive a signal necessary for differentiation.

In order to determine if the failure to receive the appropriate differentiation signal led to cell death, the thymi from the dystrophic mice and their controls were examined for apoptotic cells (Fig. 4). An increase in apoptotic thymocytes was found in the *dy/dy* mice primarily in the capsular and subcapsular regions where immature thymocytes are known to localize, We next examined each of the four subpopulations of DN thymocytes, as defined by CD44 and CD25 expression, for apoptosis using a flow cytomet-

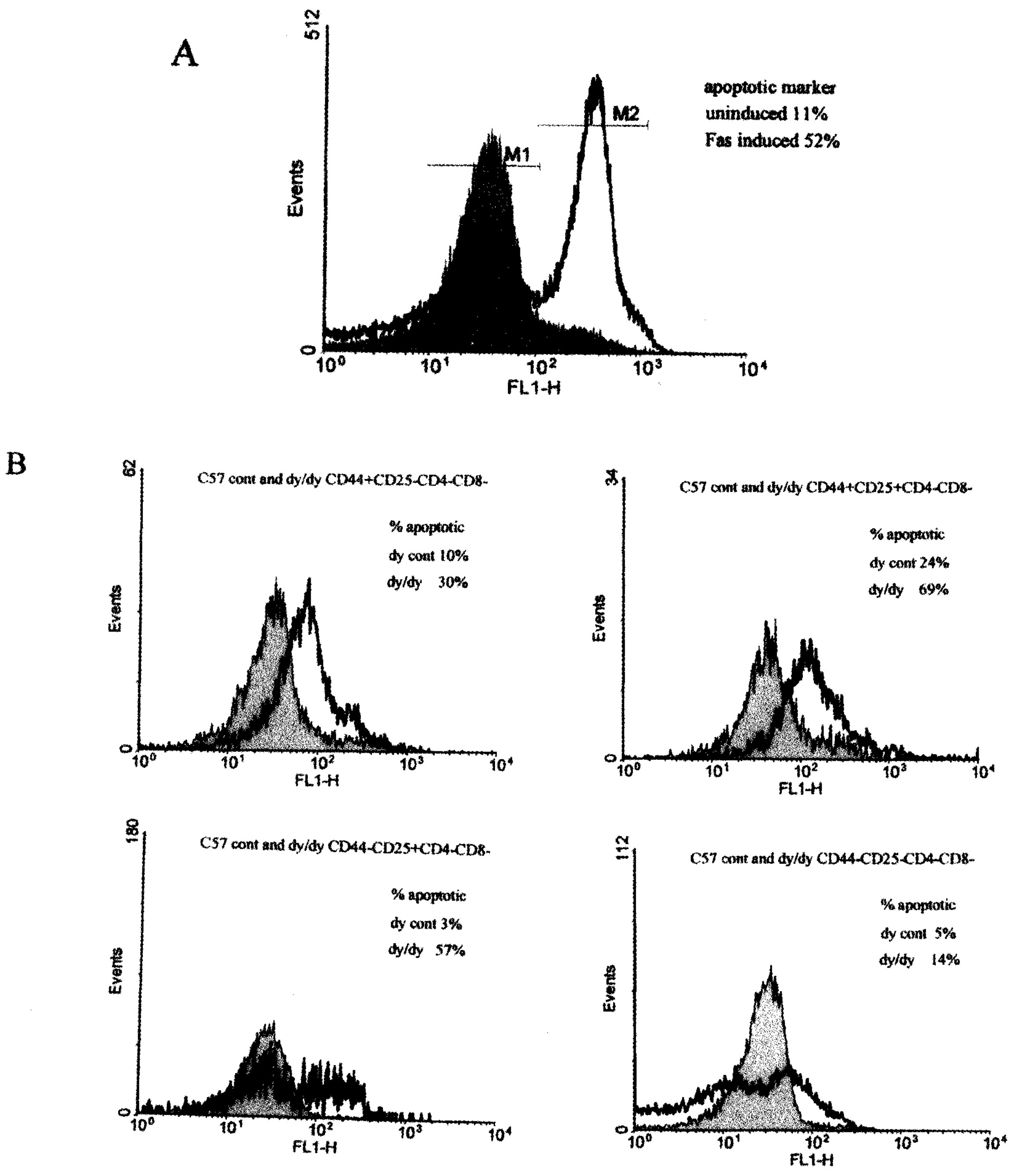

FIGURE 5 Combined flow cytometry and flow cytoenzymology define apoptotic thymocyte populations. To characterize apoptotic thymocyte populations, thymocytes isolated from dystrophic and control mice were stained with antibodies to thymic subset markers and analyzed by flow cytometry and flow cytoenzymology. The DEVD-Caspase 3 substrate (Coulter Corp.) releases a fluorophore readable on FL1 when the substrate is cleaved by caspase 3. Cell viability and surface markers were analyzed on FL2, FL3, and FL4 by typical flow cytometric techniques. A. This flow cytoenzymology histogram demonstrates the fluorescence shifts which can be obtained with this substrate. The solid peak to the left represents autofluorescence of cells loaded with substrate but not subjected to apoptotic stimulation. The peak to the right was obtained from cells stimulated with crosslinked anti-Fas antibodies 3 hours prior to exposure to substrate. This control was used to set markers by which to define the percent of apoptotic cells. B. DN thymocytes were prepared from *dy/dy* and *dy* control mice and stained with PE-anti-CD44 and biotinyl-anti-CD25 plus streptavidin-APC to obtain profiles (FL2 vs FL4) similar to Figure 2B. The subpopulations were then analyzed for caspase activity in FL1. The histograms shown here display *dy* contol profiles in filled peaks with *dy/dy* profiles overlaid. The percent of cells shifted into the apoptotic markers is noted in each panel ric assay. Each of the populations exhibited a significant increase in apoptotic cells relative to control thymocytes. This indicates that, at each stage of differentiation in the DN compartment of the thymus, laminin-2 is required for survival of the majority of cells or that failure to receive a laminin-2 survival signal early in differentiation programs thymocytes for death.

To determine the stage of development at which laminin-2 plays it's critical role, we examined thymocyte profiles from fetal day 16 through neonatal day 14. Thymocytes from these animals were analyzed by FACS for CD4, CD8, CD25 and CD44 expression while other tissues were analyzed by PCR for genotype and gender determination (data not shown). In the ages analyzed, we did not observe any significant perturbation of thymocyte subsets. It appears that laminin-2 is required for normal thymocyte development as mice reach young adulthood (2–4 weeks of age).

We examined the B cell, NK cell and γδ T cell populations by flow cytometry, taking advantage of a variety of cell surface markers, but did not find any perturbation of any of these populations. Thus, in the immune system, the developmental effects of the lack of laminin-2 appear to be restricted to specific early αβ T cell progenitors.

We used several strategies to test the function of positive and negative selection in these mice (data not shown). Immunoglobulin class switching occurred normally in the *dy/dy* mice and their response to ovalbumin was not different from control littermates. Our examinations of the T cell receptor repertoires and primary responses of thymocytes and $Ia^-$splenocytes have not revealed any alterations in the *dy/dy* mice. Therefore, we have concluded that both positive and negative selection appear to be functioning normally in these mice. The loss of thymocytes of specific types without a gross disruption of mature T cell repertoire or response may be taken as an indication that the developmental role of laminin-2 is related to proliferation or survival without affecting T cell differentation (Guidos, 1996). The ratio of DP to SP thymocytes in *dy/dy* mice relative to dy control mice suggests that there may be alterations in selection, proliferation or emigration which were not detected by our assays. These intriguing possibilities can be investigated through breeding with TCR transgenic strains and will be the subject of future studies.

To assess the ability of *dy/dy* thymocytes to proliferate, thymocytes and $Ia^-$ splenocytes from *dy/dy* and *dy* control mice were compared in standard tritium incorporation assays with anti-TCR stimulation with and without co-stimulation by laminin-2, In each case, the mutant and wild type thymocytes gave indistinguishable levels of proliferation (data not shown). The lack of laminin-2 during development does not affect the proliferation of the populations tested.

We propose a model of thymocyte development (Figure 6, model 1) in which laminin-2 provides a survival signal to early thymocytes in the subcapsular region of the thymus. In the absence of this signal, thymocytes enter apoptosis while, in the presence of laminin-2, these cells are able to undergo differentiation and proliferation and further populate the thymus. Early in thymocyte development, when the cells are undergoing a rapid expansion, laminin-2 provides a signal for the developing thymocytes to survive and maintain their proliferative state. In mice lacking laminin-2, these cells instead revert to an apoptotic death program and a major portion of the thymocytes do not survive to populate the thymus. The dramatic increase in apoptosis evident in the capsule and subcapsular regions of the *dy/dy* thymi appears sufficient to explain the overall decrease in the number of thymocytes by showing that most cells in the immature thymocyte population are in the process of dying. This would result in fewer thymocytes entering the DP population which would explain why cortical regions are not readily defined by H&E staining despite the presence of cortical epithelium.

An alternative model (Figure 6, model 2) would have laminin-2 deliver a proliferative signal to early thymocytes. In the case of the *dy/dy* mice, this signal would be lacking and thymocytes would proceed to develop and migrate but would not proliferate. This model would explain the diminished thymocyte numbers in all of the CD4/CD8 thymic populations but does not readily explain the decrease of specific pop-

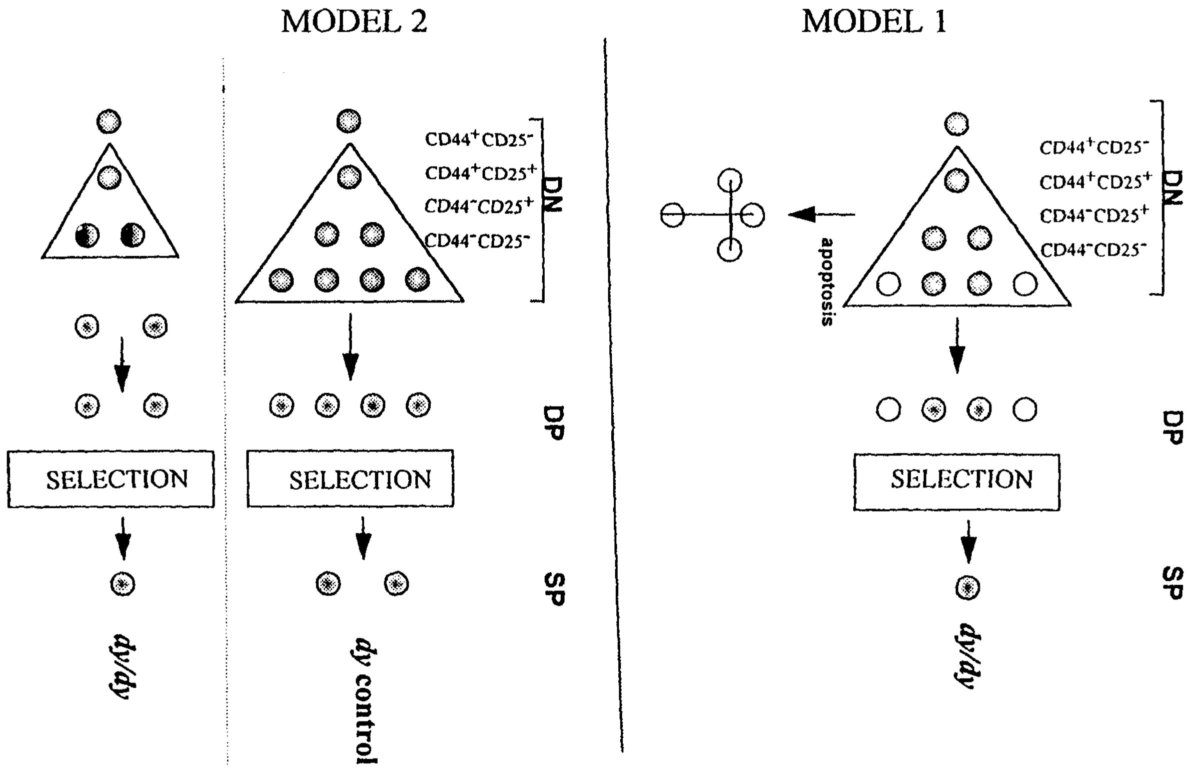

FIGURE 6 Models to explain the effect of the *dy* mutation on thymocyte development. Model 1 -- we propose that laminin-2 provides a survival signal needed by thymocytes at specific developmental checkpoints in their early maturation. The absence of this signal results in increased cell death early in development and thus a lack of cells moving along the thymic maturation pathway. Specific populations which have a more stringent requirement for this signal may then be underrepresented relative to other populations. Lack of a survival signal would be predicted to lead to an increase in programmed cell death among the populations which require the signal. Model 2 -- alternatively, we propose that laminin-2 could provide a proliferative signal to the developing thymocytes. The loss of this signal would diminish the number of cells progressing along the route to maturation. This model would affect overall cell number and could result in underrepresentation of specific populations in which this proliferative signal was required. This model would not predict an alteration in programmed cell death ulations. This model would also not explain an increase in apoptotic death of immature thymocytes.

We cannot differentiate between the specific survival signal described in our first model and the possibility that laminin-2 may be required to direct or stabilize thymocytes to receive positive selection signals. In such a case, the lack of laminin-2 could result in "death by neglect" for the majority of thymocytes. For this reason, these two possibilities are considered indistinguishable and both covered by this model In support of the concept of a specific survival signal, Vachon *et al* (Vachon et al, 1996) describe the role of merosin (laminin-2) in myogenesis as "to promote myotube stability by preventing apoptosis". In many ways, the increased apoptosis seen in the myogenesis system and the protective effect of merosin parallel our results with thymocytes from laminin-2 deficient mice.

Our study of the $dy^{2J}$ mice indicates that their specific mutation in laminin-2 produced comparable physical symptoms without the concomitant loss of thymocytes. Our analyses of the $dy^{2J}$ mice support our conclusions from studying the *dy/dy* mice by demonstrating that the thymocyte loss does not correlate with the disease -- the two strains show comparable physical symptoms but the thymic effects are only found in the strain which does not express laminin-2. It is also apparent from this result that the N terminal region of the α2 chain of laminin is not critical for laminin-2 function in the thymus but it is critical in muscle. This can be explained by our hypothesis of the role of VLA-6 in the thymus and the reported role of α-dystroglycan in muscle.

Our *in vitro* studies of thymocyte binding to laminin-2 demonstrated that the cellular receptor for laminin-2 was the integrin VLA-6 ($\alpha_6\beta_1$) (Chang et al, 1993). Studies of the dystrophin-associated glycoprotein complex in muscle showed that its component α-dystroglycan was the laminin-2 binding protein (Worton, 1995). Since the defect in $dy^{2J}$ mice, which leads to muscular dystrophy symptoms, has been mapped as an N-terminal laminin α2 deletion, and yet thymocytes from these mice undergo normal development, we can conclude that the α-dystroglycan binding site and the $\alpha_6\beta_1$ binding site are distinct. This agrees with a previous study (Deutzmann et al, 1990) in which the $\alpha_6\beta_1$ binding site was mapped to a region outside of the N terminal segment deleted in the $dy^{2J}$ mice.

The dystrophic mice have provided an *in vivo* system in which to directly examine the effects of the loss of a specific extracellular matrix protein. Our earlier hypothesis (Chang et al, 1993) of a role for laminin-2 and VLA-6 in thymocyte development has now been demonstrated directly and this function has been specifically identified as a mediator of cell survival (Giancotti, 1997, Meredith et al, 1993). This extracellular matrix protein thus is delivering a specific signal to a select population of cells at a critical point in their development. The lack of this signal then results in the activation of a default programmed cell death pathway. The resultant apoptosis causes a diminution of the immature thymocytes available to progress through development, maturation and selection leading to fewer thymocytes and T cells to populate the animal.

### *Acknowledgements*

The authors would like to thank Drs. Edgar Fernandez, Jorge Ochoa-Garay and Ken Parker for their helpful discussions and critical review of this manuscript.

### *References*

Berliner, E., Smith, J.H., Kuylen, N.B., Galiounghi, A. and Lucas, F.J. (1997). Flow cytoenzymology as a tool for rapid cell-type and cell-lineage identification of red blood cells and blast cells. *Lab. Haemat.* **3**: 72–77.

Brown, C., Sauvageot, J., Kahane, H. and Epstein, J. I. (1996). Cell proliferation and apoptosis in prostate cancer -- Correlation with pathologic stage? *Mod. Pathol.* **9**: 205–209.

Burgeson, R. E., Chiquet, M., Deutzmann, R., Ekblom, P., Engel, J., Kleinman, H., Martin, G. R., Meneguzzi, G., Paulsson, M., Sanes, J., Timpl, R., Tryggvason, K., Yamada, Y., and Yurchenco, P. D. (1994). A new nomenclature for the laminins. *Matrix Biology*. **14**: 209–211.

Campbell, K.P. (1995). Three muscular dystrophies: loss of cytoskeleton-extracellular matrix linkage. *Cell* **80**: 675–679.

Chang, A. C., Wadsworth, S. and Coligan, J. E. (1993). Expression of laminin-2 in the thymus and its interaction with thymocytes. *J. Immunol.* **151**: 1789–1801.

Chang, A. C., Salomon, D. R., Wadsworth, S., Hong, M-J. P., Mojcik, C. F., Otto, S., Shevach, E. M. and Coligan, J. E. (1995). $\alpha_3\beta_1$ and $\alpha_6\beta_1$ integrins mediate laminin/merosin binding and function as costimulatory molecules for human thymocyte proliferation. *J. Immunol.* **154**: 500–510.

Deutzmann, R., Aumailley, M., Wiedemann, H., Pysny, W., Timpl, R. and Edgar, D. (1990). Cell adhesion, spreading and neurite stimulation by laminin fragment E8 depends on maintanence of secondary and tertiary structure in its rod and globular domain. *Eur. J. Biochem.* **191**: 513–522.

Duowitz, V. (1992). The muscular dystrophies. *Postgrad. Med. J.* **68**: 500–506.

Ehrig, K., Leivo, I., Argraves, W. S., Ruoslahti, E. and Engvall, E. (1990). Merosin, a tissue-specific basement membrane protein, is a laminin-like protein. *Proc. Natl. Acad. Sci. USA.* **87**: 3264–3268.

Fassler, R. and Meyer, M. (1995). Consequences of lack of beta 1 integrin gene expression in mice. Genes Dev. **9**: 1896–1908.

Giancotti, F. G. (1997). Integrin signaling: Specificity and control of cell survival and cell cycle progression. *Curr. Opin. Cell Biol.* **9**: 691–700.

Godfrey, D. I., Kennedy, J., Suda, T., and Zlotnik, A. (1993). A developmental pathway involving four phenotypically and functionally distinct subsets of CD3-CD4-CD8- triple-negative adult mouse thymocytes defined by CD44 and CD25 expression. *J. Immunol.* **150**: 4244–4252.

Godfrey, D. I., Kennedy, J., Mombaerts, P., Tonegawa, S. and Zlotnik, A. (1994). Onset of TCR-β gne rearrangement and role of TCR-β expression during CD3-CD4-CD8-thymocyte differentiation. *J. Immunol.* **152**: 4783–4792.

Guidos, C. J. (1996). Positive selection of CD4+and CD8+ T cells. *Curr. Opin. in Immunol.* **8**: 225–232.

Hynes, R. O. (1992). Integrins: Versatility, modulation, and signaling in cell adhesion. *Cell.* **69**: 11–25.

Lannes-Vieira, J., Chammas, R., Villa-Verde, D. M. S., Vannier-dos-Santos, M. A., Mello-Coelho, V., de Souza, S. J., Brentani, R. R. and Savino, W. (1993). Extracellular matrix components of the mouse thymic microenvironment. III. Thymic epithelial cells express the VLA6 complex that is involved in laminin-mediated interaction with thymocytes. *International Immunology*. **5**: 1421–1430.

Lannes-Vieira, J., Dardenne, M. and Savino, W. (1990). Extracellular matrix components of the mouse thymus microenvironment: ontogenetic studies and modulation by glucocorticoid hormones. *J. Histochem. Cytochem.* **39**: 1539–1546.

Leivo, I., and Engvall, E. (1988). Merosin, a protein specific for basement membranes of Schwann cells, striated muscle, and trophoblast, is expressed late in nerve and muscle development. *Proc. Natl. Acad. Sci. USA.* **85**: 1544–1548.

Malik, R.K. (1997). Regulation of apoptosis by integrin receptors. *J. Ped. Hemat./Oncol.* **19**: 541–545.

Matsumura, K., and Campbell, K. P. (1993). Deficiency of dystrophin-associated proteins: a common mechanism leading to muscle cell necrosis in severe childhood muscular dystrophies. *Neuromusc. Disord.* **3**: 109–118.

Meredith, J. E. Jr., Fazeli, B., Schwartz, M.A. (1993). The extracellular matrix as a cell survival factor. *Mol. Biol. Cell.* **4**: 953–961.

Porter, A. G. and Janicke, R.U. (1999). Emerging roles of caspase-3 in apoptosis. *Cell Death Differ.* **6**: 99–104.

Ritter, M. A., and Boyd, R. L. (1993). Development in the thymus: it takes two to tango. *Immunol. Today.* **14**: 462–469.

Ruiz, P., Wiles, M.V., and Imhof, B.A. (1995). $\alpha_6$ integrins participate in pro-T cell homing to the thymus. *Eur. J. Immunol.* **25**: 2034–2041.

Savino, W. and Silva-Barbosa, S.D. (1996). Laminin/VLA-6 interactions and T cell function. *Braz. J. Med. Biol. Res.* **29**: 1209–1220.

Scollay, R., and Godfrey, D. I. (1995). Thymic emigration: conveyor belts or lucky dips? *Immunol. Today.* **16**: 268–273.

Shimizu, Y., van Seventer, G. A., Horgan, K. J. and Shaw, S. (1990). Regulated expression and binding of three VLA ($\beta_1$) integrin receptors on T cells. *Nature.* **345**: 250–253.

Sprent, J., Lo, D., Gao, E.-K. and Ron, Y. (1988). T cell selection in the thymus. *Immunol. Rev.* **101**: 173–190.

Stephens, L. E., Sutherland, A.E., Klimanskaya, I.V., Andrieux, A., Meneses, J., Pedersen, R. A. and Damsky, C. H. (1995). Deletion of beta 1 integrins in mice results in inner cell mass failure and peri-implantation lethality. *Genes Dev.* **9**: 1883–1895.

Sunada, Y., Bernier, S. M., Kozak, C. A., Yamada, Y. and Campbell, K. P. (1994). Deficiency of merosin in dystrophic *dy* mice and genetic linkage of laminin M chain gene to *dy* locus. *J. Biol. Chem.* **269**: 13729–13732.

Sunada, Y., Bernier, S. M., Utani, A., Yamada, Y. and Campbell, K. P. (1995). Identification of a novel mutant transcript of laminin $\alpha 2$ chain gene responsible for muscular dystrophy and dysmyelination in *dy*2J mice. *Human Molecular Genetics.* **4**: 1055–1061.

Vachon, P. H., Loechel, F., Xu, H., Wewer, U. M. and Engvall, E. (1996). Merosin and laminin in myogenesis; specific requirement for merosin in myotube stability and survival. *J. Cell Biol.* **134**: 1483–1497.

Wadsworth, S, Halvorson, M. J., Chang, A. C. and Coligan, J. E. (1993). Multiple changes in VLA protein glycosylation, expression, and function occur during mouse T cell ontogeny. *J. Immunol.* **150**: 847–857.

Wewer, U. M. and Engvall, E. (1994). Laminins. *Methods in Enzymology* **245**: 85–104.

Wewer, U. M., Wayner, W. A., Hoffstrom, B. G., Lan, F., Meyer-Nielsen, B., Engvall, E., and Albrechtsen, R. (1994). Selective assembly of laminin variants by human carcinoma cells. *Laboratory Investigation.* **71**: 719–730.

Worton, R. (1995). Muscular dystrophies: Diseases of the dystrophin-glycoprotein complex. *Science.* **270**: 755–6.

Xu, H., Wu, X-R., Wewer, U. M. and Engvall, E. (1994). Murine muscular dystrophy caused by a mutation in the laminin $\alpha 2$ (Lama2) gene. *Nature Genetics* **8**: 297–302.

Xu, H., Christmas, P., Wu, X.-R., Wewer, U. M. and Engvall, E. (1994). Defective muscle basement membrane and lack of M-laminin in the dystrophic *dy/dy* mouse. *Proc. Natl. Acad. Sci. USA.* **91**: 5572–5576.

